# Chk2-p53 and JNK in irradiation-induced cell death of hematopoietic progenitors and differentiated cells in *Drosophila* larval lymph gland

**DOI:** 10.1242/bio.058809

**Published:** 2021-08-23

**Authors:** Tram Thi Ngoc Nguyen, Jiwon Shim, Young-Han Song

**Affiliations:** 1 Department of Biomedical Gerontology, Hallym University, Chuncheon, Gangwon-do 24252, Republic of Korea; 2 Ilsong Institute of Life Science, Hallym University, Seoul 07247, Republic of Korea; 3 Department of Life Science, College of Natural Science, Hanyang University, Seoul 04763, Republic of Korea

**Keywords:** Ionizing radiation, *Drosophila*, Hematopoietic progenitor, Hematopoiesis, Cell death, DNA damage response

## Abstract

Ionizing radiation (IR) induces DNA double-strand breaks that activate the DNA damage response (DDR), which leads to cell cycle arrest, senescence, or apoptotic cell death. Understanding the DDR of stem cells is critical to tissue homeostasis and the survival of the organism. *Drosophila* hematopoiesis serves as a model system for sensing stress and environmental changes; however, their response to DNA damage remains largely unexplored. The *Drosophila* lymph gland is the larval hematopoietic organ, where stem-like progenitors proliferate and differentiate into mature blood cells called hemocytes. We found that apoptotic cell death was induced in progenitors and hemocytes after 40 Gy irradiation, with progenitors showing more resistance to IR-induced cell death compared to hemocytes at a lower dose. Furthermore, we found that *Drosophila ATM* (*tefu*), *Chk2* (*lok*), *p53*, and *reaper* were necessary for IR-induced cell death in the progenitors. Notably, IR-induced cell death in mature hemocytes required *tefu*, *Drosophila JNK (bsk)*, and *reaper*, but not *lok* or *p53*. In summary, we found that DNA damage induces apoptotic cell death in the late third instar larval lymph gland and identified *lok*/*p53*-dependent and -independent cell death pathways in progenitors and mature hemocytes, respectively.

## Abstract (precis)

**Summary:** Irradiation-induced cell death in larval lymph gland requires *Drosophila* ATM, Chk2, p53, and reaper in hematopoietic progenitors, while it involves *Drosophila* ATM, JNK, and reaper in differentiated blood cells.

## INTRODUCTION

Cellular DNA is damaged by endogenous insults such as reactive oxygen species (ROS) generated during cell metabolism and exogenous genotoxic agents, including ionizing radiation (IR) (Song, 2005). Damaged DNA activates the DNA damage response (DDR), resulting in cell cycle arrest, DNA repair, senescence, and apoptotic cell death. The fate of DNA-damaged cells depends on the severity and nature of the DNA damage, as well as the genetic status and type of cells. Understanding the DDR of stem cells is important because proper maintenance of tissue homeostasis during normal development is critical for the survival of the organism. Moreover, it will provide insights into the effective killing of cancer stem cells during anticancer therapy because the cancer stem cells share similar properties with stem cells, including unlimited proliferative potential and self-renewal.

In humans, DNA damage activates the protein kinase ATM stimulating downstream kinases Chk1 and Chk2. Chk1 and Chk2 phosphorylate and stabilize p53, which acts as a transcription factor to induce genes involved in cell cycle arrest and apoptosis. Since the discovery of the *p53* ortholog (Ollmann et al., 2000; Brodsky et al., 2000), a key regulator of DDR, *Drosophila* has served as a model system for studying DDR (Song, 2005). The genes involved in the DDR are conserved in *Drosophila* and DNA damage-induced cell death occurs through *tefu* (*Drosophila* ATM) (Song et al., 2004), *lok* (*Drosophila* Chk2) (Brodsky et al., 2004; Peters et al., 2002; Xu and Du, 2003), and *p53* (*Drosophila* p53) (Brodsky et al., 2004; Dichtel-Danjoy et al., 2013). *Drosophila p53* induces proapoptotic genes including *hid*, *reaper*, or *grim* (Brodsky et al., 2000, 2004; Moon et al., 2008). Although studies in *Drosophila* have helped us make considerable progress in our knowledge of stem cell biology, the DDR in *Drosophila* stem cells, especially hematopoietic stem cells, is relatively less explored.

*Drosophila* hematopoiesis occurs in two distinct locations during different developmental stages (Banerjee et al., 2019). The first embryonic phase originates in the head mesoderm, producing both circulating and sessile pools of blood cells, called hemocytes, that persist into the adult stage. The second wave occurs during larval development in a hematopoietic organ, the lymph gland. In the third instar larvae (3L), the mature lymph gland comprises a pair of anterior primary lobes that are formed during embryogenesis and a variable number of more posterior secondary lobes (Yu et al., 2018). The primary lobe, which is the best characterized among the anterior and posterior lobes, consists of a medullary zone (MZ), cortical zone (CZ), and the niche, called the posterior signaling center (Fig. S1A). The MZ, located in the core, contains hematopoietic progenitors that differentiate into mature hemocytes in the outermost region, CZ. Hematopoietic progenitors are considered stem-like cells because of their ability to differentiate into various myeloid-type blood cells. The *Drosophila* hematopoietic system responds to internal and external stresses, and the mechanism for this regulation has been well established. However, the DDR in the lymph gland remains unknown.

To understand the DDR of hematopoietic progenitors, the mechanism and cellular response after irradiation in the lymph gland of 3L were investigated and compared to those of differentiated hemocytes. We found that both hematopoietic progenitors and differentiated cells undergo apoptotic cell death upon 40 Gy irradiation, while progenitors are more resistant to cell death than differentiated hemocytes at lower dose irradiation. IR-induced cell death in progenitors occurs through the canonical DDR pathway, *tefu*-*lok*-*p53*-*reaper*. On the other hand, cell death in the differentiated hemocytes was *lok*- and *p53*-independent, requiring *Drosophila* JNK, *bsk*.

## RESULTS AND DISCUSSION

### IR induces cell death of hematopoietic cells at the 3L stage

**Fig. 1.**
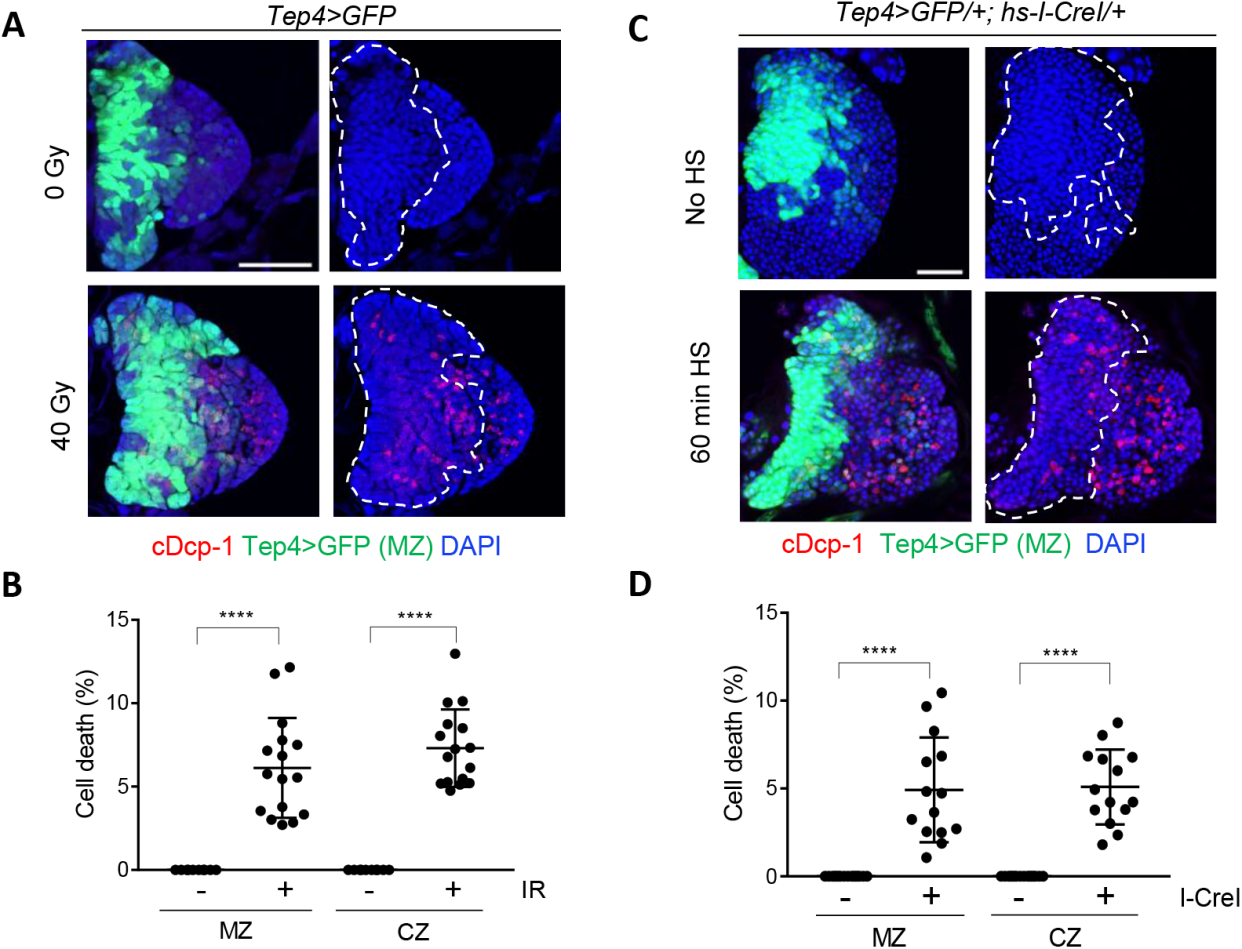
**IR induces cell death in the lymph gland of *Drosophila* third instar larvae (3L).** The 3L were irradiated at 40 Gy (A,B) or heat-shock treated to overexpress *I-CreI* (C,D). Four hours after treatment, the lymph gland was stained with cDcp-1 antibody. Scale bars: 50 µm. DAPI (blue), Tep4>GFP (green), and cDcp-1 (red) indicate DNA, progenitors, and apoptotic cells, respectively. The boundary of the Tep4>GFP-stained MZ is marked with white dotted lines. (B,D) Percentages of cell number with cDcp-1 signal in progenitors (MZ) and differentiated cells (CZ) for indicated genotypes in (A) and (C) with (+) and without (−) treatment (IR or I-CreI) are shown. ****P<0.0001.

To test the cellular response of hematopoietic cells in response to DNA damage, 3L expressing GFP in MZ by *Tep4* promoter (*Tep4-Gal4*, *UAS-GFP*, indicated as *Tep4>GFP*) (Fig. S1B) were irradiated and stained with the apoptosis marker, active cleaved Dcp-1 (*Drosophila* Caspase) (cDcp-1). In the absence of irradiation, cDcp-1 was not detected (Fig. 1A, upper panel). Four hours after irradiation, the cDcp-1 signal was increased in both *Tep4>GFP*-positive MZ (6.1%) and *Tep4>GFP*-negative CZ (7.3%) (Fig. 1B). TUNEL staining was also increased after irradiation (Fig. S2), confirming that IR induced apoptotic cell death in both progenitors and differentiated hemocytes in the 3L lymph gland. *Drosophila* hematopoiesis is affected by the cell death-induced loss of MZ or CZ (Dey et al., 2016; Mondal et al., 2011). Despite the increase in cDcp-1 or TUNEL signal, DNA damage did not change the size of the primary lobe and the proportion of MZ (Fig. S3), presumably because these phenotypes were observed shortly (4 h) after DNA damage induction.

Since the most significant consequence of IR in the cells is the generation of DNA double-strand breaks (DSBs), we stained the irradiated lymph gland with antibody that specifically recognizes phosphorylated histone His2Av (γ-His2Av). His2Av, the *Drosophila* ortholog of histone H2AX, is rapidly phosphorylated in the vicinity of DSBs (Lake et al., 2013). In the absence of irradiation, the intensity of γ-His2Av staining was undetectable in both the MZ and CZ (Fig. S4A, upper panel, B). One hour after irradiation, the γ-His2Av signals were similarly increased in both progenitors in the MZ and differentiated cells in the CZ (Fig. S4A, lower panel, B), suggesting comparable DNA repair kinetics of DSBs in these compartments. To confirm that cell death was induced by DNA damage rather than by other types of cellular damage, DSBs were generated using endonuclease *I-CreI*. *I-CreI* generates DSBs in 18S ribosome gene repeats, and overexpression of *I-CreI* using the heat-shock promoter (*hs-I-CreI*) has been used to induce DDR in *Drosophila* (Ma et al., 2016). Heat-shock of *Tep4>GFP* larvae (Fig. S5) or *Tep4>GFP/+; hs-I-CreI/+* larvae without heat-shock (Fig. 1C, upper panel) did not induce cell death. *I-CreI* expression by 60 min of heat-shock induced cell death (Fig. 1C, lower panel) in both MZ (4.9%) and CZ (5.1%) (Fig. 1D), confirming that the DNA damage caused cell death of hematopoietic progenitors and differentiated hemocytes in the 3L lymph gland.

### Sensitivity to DNA damage-induced cell death in hematopoietic progenitors and the differentiated hemocytes

**Fig. 2.**
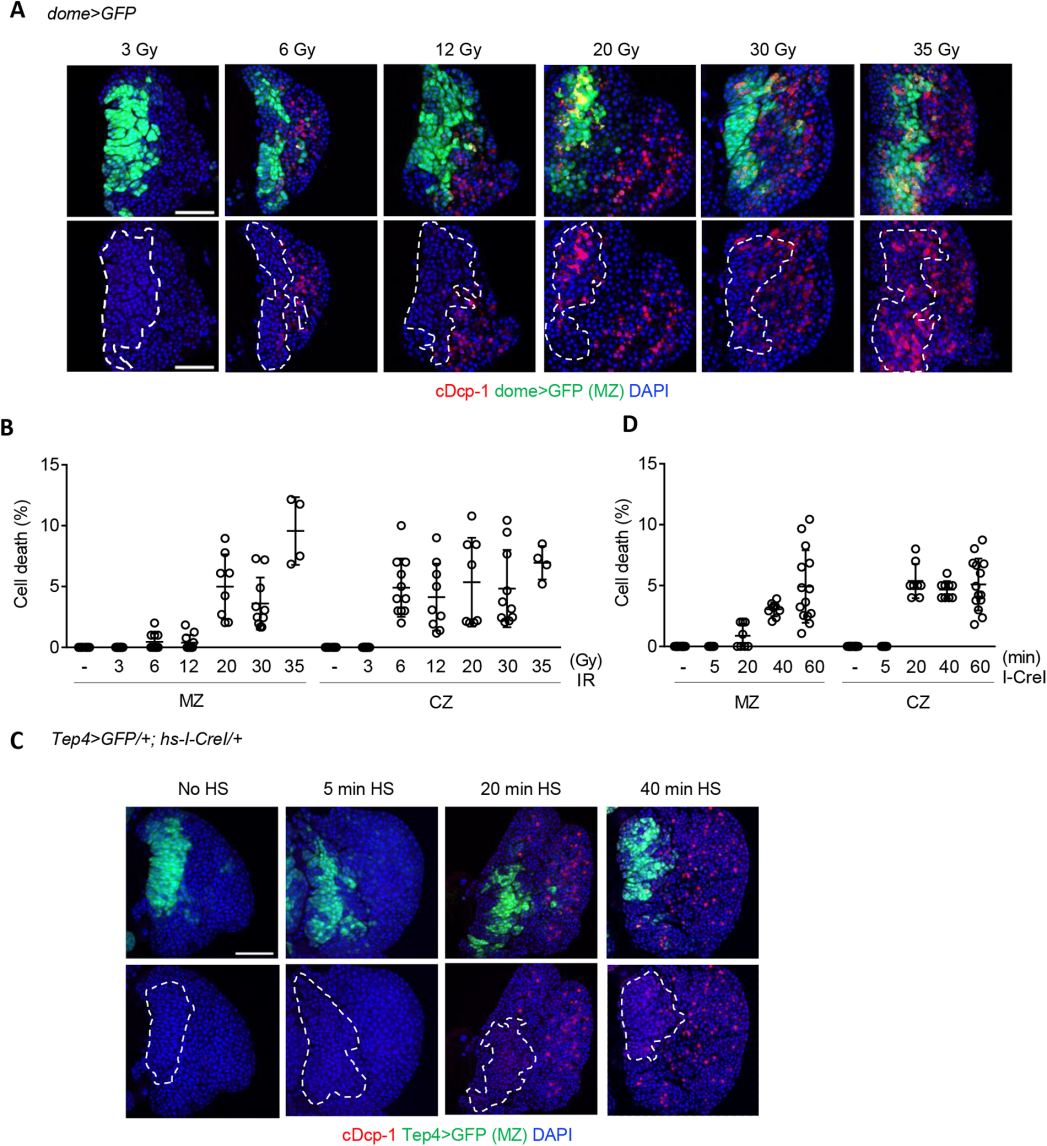
**Progenitors in the 3L lymph gland are more resistant to DNA damage-induced cell death than the differentiated hemocytes.** The 3L were irradiated at indicated doses (A,B) or heat-shock treated (HS) for the indicated time (C,D). At 4 h after treatment, cDcp-1 staining was performed using the lymph glands. The boundaries of MZ marked by dome>GFP (A) or Tep4>GFP (C) are indicated with white dotted lines. (B,D) Percentages of cell number with cDcp-1 signal in progenitors (MZ) and differentiated cells (CZ) after treatment are shown. Scale bars: 50 µm.

To compare the sensitivity of DNA damage-induced cell death between hematopoietic progenitors and their differentiated cells, lower amounts of DNA damage were generated by decreasing the doses of irradiation or duration of heat-shock. In the *dome>GFP*-negative CZ, cell death was detected at doses of 6 Gy or higher (Fig. 2A,B). On the other hand, irradiation at 20 Gy or higher was required to induce cell death in the progenitor cells in the MZ (Fig. 2A,B). Similarly, induction of *hs-I-CreI* by 20 min heat-shock increased cell death in the differentiated hemocytes in CZ, while it resulted in very few, if any, dying cells in the progenitors (Fig. 2C,D). These results suggest that the progenitor cells in the 3L lymph gland are more resistant to DNA damage-induced cell death than the differentiated hemocytes. DNA damage-induced apoptotic response is repressed by cell cycle arrest at either G1/S or G2/M in *Drosophila* oogenesis (Qi and Calvi, 2016). The progenitors in the 3L are arrested in the G2 phase of the cell cycle (Sharma et al., 2019), while mature hemocytes in 3L CZ are proliferating (Jung et al., 2005; Krzemien et al., 2010); suggesting that cell cycle profile may affect the radiation sensitivity of these cells.

Similar to other *Drosophila* adult stem cells in the germline and midgut (Xing et al., 2015), hematopoietic progenitors were more resistant to IR-induced cell death than their differentiated hemocytes. However, apoptotic cell death in hematopoietic progenitors can be detected upon 20 Gy irradiation (Fig. 2B), which is in contrast to other *Drosophila* stem cells that survive high-dose irradiation up to 50 Gy (Xing et al., 2015; Wagle and Song, 2020). In general, stem cells possess various cytoprotective properties to maintain tissue homeostasis throughout life (Seita et al., 2010). For example, many adult stem cells are in a quiescent state, which minimizes DNA damage due to replication errors (Fuchs and Horsley, 2011; Wilson et al., 2008). Moreover, stem cells generate energy predominantly via the glycolytic pathway rather than mitochondrial respiration, thus maintaining lower levels of ROS, which may reduce DNA damage (Mandal et al., 2011). The radiation sensitivity of hematopoietic progenitors compared to other stem cells could be because they are not ‘classic’ stem cells, as evident by a lack of asymmetric cell division, which is a hallmark of stem cells (Krzemien et al., 2010). Alternatively, they have unique features that may explain their sensitivity to radiation-induced cell death. For example, the progenitors in the 3L contain a high basal level of ROS functioning as a differentiation signal (Owusu-Ansah and Banerjee, 2009), which may sensitize these cells to IR-induced cell death. The mechanisms underlying the decision between survival and death in different *Drosophila* stem cells are currently under investigation.

### *Drosophila* ATM, Chk2, p53, and reaper are required for IR-induced cell death in the hematopoietic progenitors in the 3L lymph gland

**Fig. 3.**
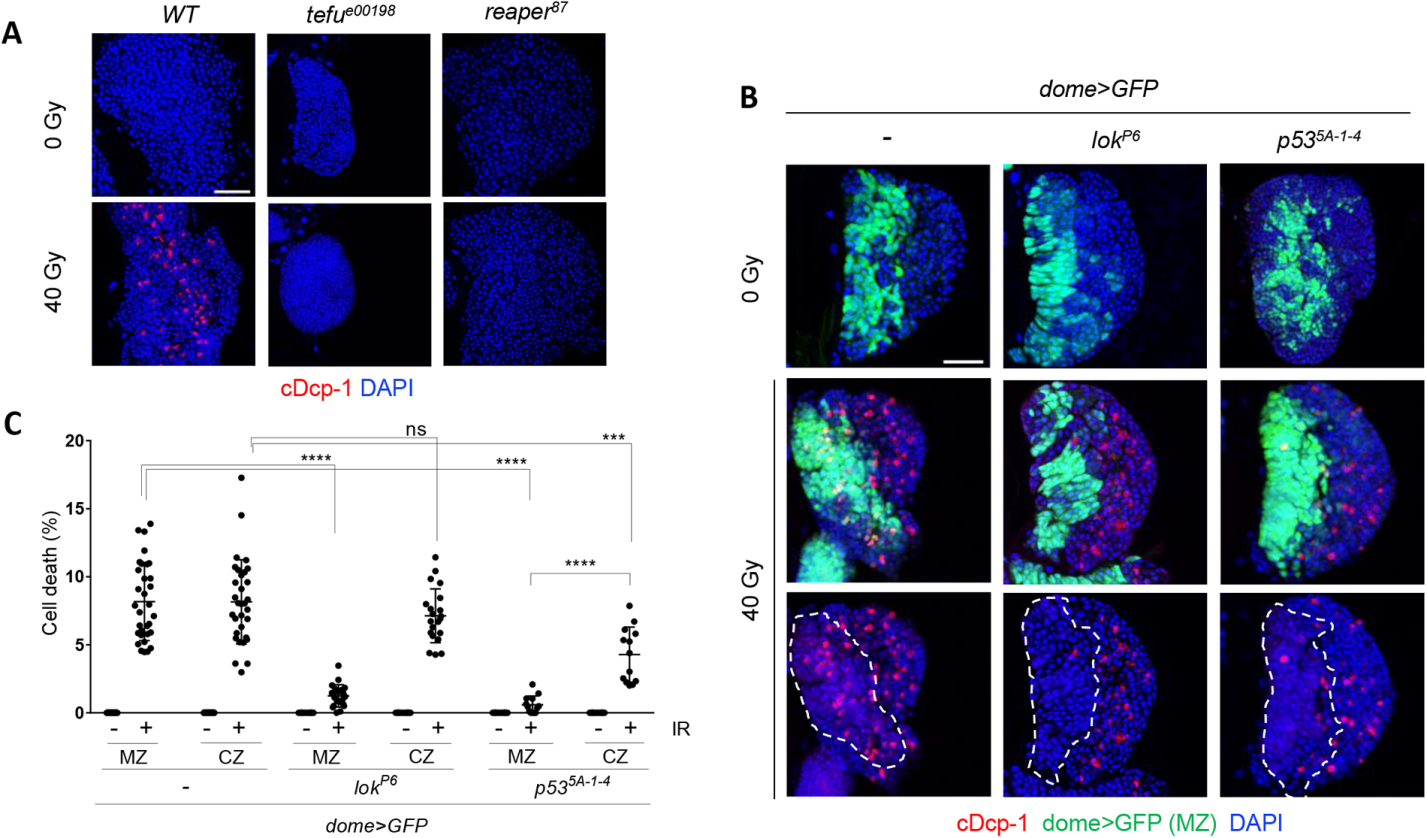
**Genes involved in IR-induced cell death in the 3L lymph gland.** (A,B) The 3L with indicated genotype were irradiated at 40 Gy, and the lymph gland was stained using cDcp-1 antibody 4 h post-irradiation. The MZ was marked with dome>GFP and indicated as white dotted lines (B). DAPI (blue), dome>GFP (green), and cDcp-1 (red) indicate DNA, progenitors, and cell death, respectively. Scale bars: 50 µm. (C) Percentage of cell number with cDcp-1 signal in progenitors (MZ) and differentiated cell (CZ) in *lok^P6^ and p53^5A-1-4^* mutant with (+) or without (−) irradiation are shown. ****P<0.0001; ***P<0.001; ns, not significant.

DNA damage-induced death of mitotically dividing somatic cells in *Drosophila* requires activation of protein kinases, *tefu* (*Drosophila* ATM) and *lok* (*Drosophila* Chk2), resulting in the activation of the transcription factor *p53,* which induces the expression of pro-apoptotic genes *hid*, *reaper*, and *grim* (Song, 2005). To test whether the same genes are involved in IR-induced cell death in the 3L lymph gland, *tefu^e00198^*, *lok^P6^*, *p53^5A-1-4^*, and *reaper^87^* mutant larvae were irradiated and stained with cDcp-1. In the absence of irradiation, cDcp-1 signal was not detected in any of the mutant lymph glands (Fig. 3). After irradiation, no cell death was induced in the whole lymph gland in the *tefu^e00198^* and *reaper^87^* mutants (Fig. 3A). On the other hand, irradiated *lok^P6^* and *p53^5A-1-4^* mutant lymph glands exhibited cell death in *dome>GFP*-negative CZ, while significantly less cell death was detected in *dome>GFP*-positive MZ (cell death in MZ after irradiation; 8.2% in wild type, 1.3% in *lok^P6^*, and 0.6% in *p53^5A-1-4^*) (Fig. 3B,C). Cell death in the *p53^5A-1-4^* mutant lymph glands in the *dome>GFP*-negative CZ was less than that observed in the wild type, suggesting that *p53* may play a minor role in CZ. These results suggested that two signaling pathways are activated to induce cell death by IR in the 3L lymph gland in a *lok/p53*-dependent or -independent manner in the progenitors or differentiated cells, respectively. Several mechanisms of Chk2 inactivation have been reported in mammals, including transcriptional inhibition, dephosphorylation by protein phosphatase 2A, proteasomal degradation, and inhibitory phosphorylation by Polo-like kinase-1 (Zannini et al., 2014; Wichmann et al., 2006). Further investigation will elucidate the mechanism by which *lok* and *p53* are not necessary for IR-induced cell death in differentiated hemocytes.

In the absence of irradiation, the size of the primary lobe and the proportion of MZ or CZ in *lok^P6^*, *p53^5A-1-4^*, and *reaper^87^* mutants were similar to those in wild type (Fig. S6). On the other hand, the *tefu^e00198^* mutant showed a significantly smaller primary lobe than the wild type in the absence of irradiation (44.5% of wild type, Fig. S6C), suggesting that *tefu* plays a role during normal development of the lymph gland in addition to DNA damage-induced cell death. A small lymph gland in 3L has been reported when progenitor cells are genetically ablated by reaper expression (Dey et al., 2016). Since loss of *tefu* in the larval disc cells shows spontaneous chromosomal telomere fusion and apoptosis (Song et al., 2004), the *tefu* mutant could induce cell death in the hematopoietic progenitors, resulting in a small lymph gland, which remains to be studied.

### *Drosophila* JNK, *bsk*, acts downstream of *tefu* to induce cell death in the differentiated hemocytes in the 3L lymph gland

**Fig. 4.**
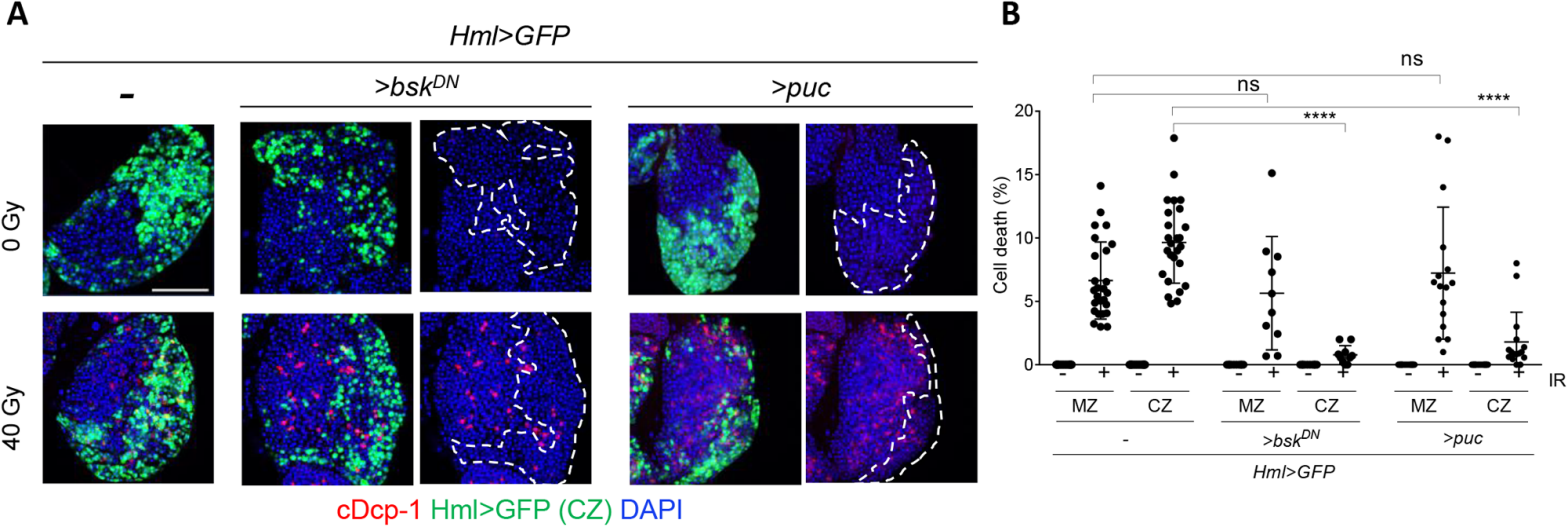
***Drosophila* JNK, *bsk*, is required for IR-induced cell death in the differentiated hemocytes.** Lymph gland from 3L expressing dominant-negative *bsk* (*UAS- bsk^DN^*) or *puckered* (*UAS-puc*) in CZ driven by *Hml-Gal4* was irradiated at 40 Gy, and the lymph glands were stained using cDcp-1 at 4 h post-irradiation. (A) Representative images of the lymph glands are shown. DAPI (blue), Hml>GFP (green), and cDcp-1 (red) indicate DNA, differentiated cells, and cell death, respectively. The boundary of CZ is marked with white dotted lines. Scale bars: 50 µm. (B) Quantitation of cell death in progenitors (MZ) and differentiated cells (CZ) before (−) and after (+) irradiation are shown. ****P<0.0001; ns, not significant.

Since *bsk* (*Drosophila* c-Jun N-terminal kinase, JNK) is required for *p53*-independent apoptosis upon irradiation (McNamee and Brodsky, 2009), we tested whether *lok*/*p53*-independent cell death in the CZ requires *bsk*. Because the null mutant of *bsk* is homozygous lethal (Sluss et al., 1996; Riesgo-Escovar et al., 1996), *bsk* activity in CZ was suppressed by overexpression of the dominant-negative form of *bsk* (*bsk^DN^*) (Zhang et al., 2015) using *Hml-Gal4* (*Hml>bsk^DN^*). In the absence of irradiation, expression of *bsk^DN^* in the CZ did not increase cell death (Fig. 4A,B). After irradiation, *bsk^DN^* expression significantly attenuated cell death in the differentiated cells in CZ (9.7% in *Hml>GFP* versus 0.8% in *Hml>GFP, >bsk^DN^*, *P*<0.0001) (Fig. 4A,B). To confirm the role of *bsk* in IR-induced cell death, we utilized a negative regulator of *bsk* signaling, JNK-specific MAPK phosphatase, *puckered* (*puc*), which can be used to efficiently block *bsk* activity when overexpressed (Martin-Blanco et al., 1998). When *puc* was expressed in CZ using *Hml-Gal4*, IR-induced cell death in CZ was significantly reduced compared to that in cells expressing only *Hml>GFP* (9.1% in *Hml>GFP* versus 1.8% in *Hml>GFP, >puc*, *P*<0.0001) (Fig. 4A,B). These results showed that *lok/p53*-independent cell death in irradiated differentiated hemocytes occurs through *Drosophila* JNK. The role of *bsk* in progenitors during IR-induced cell death remains to be studied.

In addition to the canonical DNA damage-induced cell death pathway, including *tefu*-*lok*-*p53*, *lok/p53*-independent cell death has been reported in mitotically dividing larval disc cells (Wichmann et al., 2006). When wild-type larvae are irradiated at 40 Gy, apoptosis in the wing disc, which is detected 4–30 h after irradiation, occurs in two phases (Wichmann et al., 2006, 2010). The first phase between 4–6 h is *lok/p53*-dependent and the second phase at 18 h occurs in a *lok/p53*-independent manner (Wichmann et al., 2006, 2010) and requires *bsk* (McNamee and Brodsky, 2009). Aneuploidy assayed by the *Minute* phenotype is increased after irradiation, and irradiation-induced aneuploid cells are eliminated by *bsk*-dependent and *p53*-independent cell death (McNamee and Brodsky, 2009). Additionally, *bsk*-dependent and *p53*-independent cell death has been observed under various conditions, including aneuploidy induced by loss of the spindle assembly checkpoint (Muzzopappa et al., 2017; Dekanty et al., 2012) and overexpression of histone deacetylase sir 2 (Griswold et al., 2008). Although aneuploidy is a potential cause of *p53*-independent and *bsk*-dependent cell death in CZ following IR, further investigation is required to reveal the underlying mechanism.

**Fig. 5.**
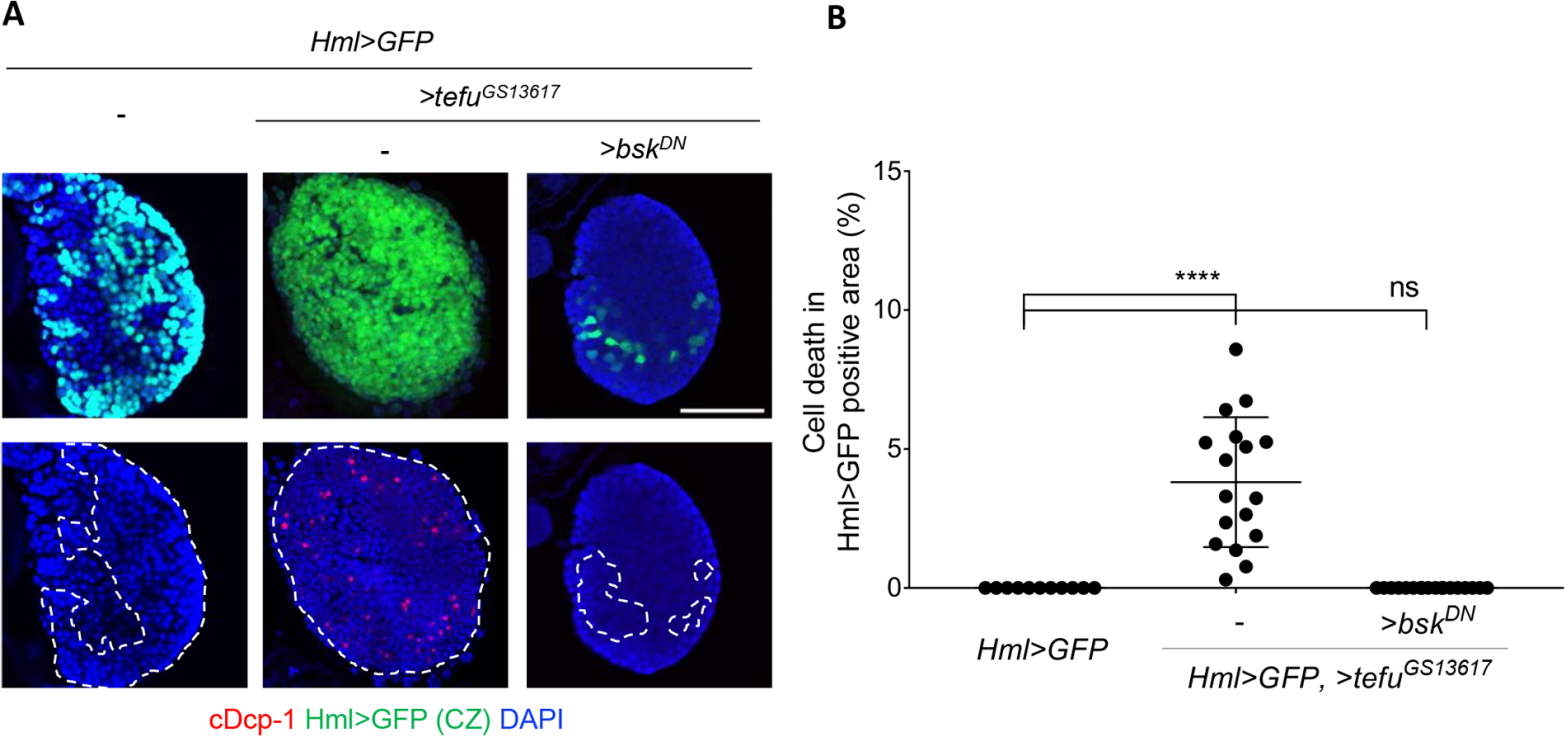
***Drosophila* JNK, *bsk*, acts downstream of *tefu* to induce cell death in the differentiated hemocytes.** The lymph glands from 3L overexpressing *tefu* in CZ (*Hml>tefu^GS13617^*) alone or together with *bsk^DN^* (*Hml>tefu^GS13617^,>bsk^DN^*) were stained using cDcp-1. (A) Representative images of the lymph glands are shown. DAPI (blue), Hml>GFP (green), and cDcp-1 (red) indicate DNA, differentiated cells, and cell death, respectively. The boundary of CZ is marked with white dotted lines. Scale bars: 50 µm. (B) Percentage of cell number with cDcp-1 signal in differentiated cells in are shown. ****P<0.0001; ns, not significant.

To determine the epistatic relationship between *tefu* and *bsk*, *tefu* was overexpressed using a mis-expression line, *tefu^GS13617^*, containing the *UAS* sequence upstream of the *tefu*-coding region (Gregory et al., 2007). When *tefu* was overexpressed in CZ using *Hml-Gal4* (*Hml>tefu^GS13617^*), cell death was induced in the differentiated cells of the 3L lymph gland in the absence of irradiation (3.8%) (Fig. 5A,B), suggesting that *tefu* overexpression was sufficient to induce cell death in these cells. The *tefu*-induced cell death was suppressed when *bsk^DN^* was co-expressed (Fig. 5A,B), suggesting that *bsk* acts downstream of *tefu* to induce cell death in the differentiated cells. The lack of cDcp-1stained cells in *tefu* and *bsk^DN^* co-expressing cells was not due to the reduction of Hml>GFP-positive cells, as cell death was detected in 3.8% of Hml>GFP-positive cells when *tefu* was overexpressed and more than thousand Hml>GFP-positive cells were observed, showing no cDCP-1 staining in *tefu* and *bsk^DN^* co-expressing cells. In support of the above data, ATM-mediated phosphorylation of JNK has been reported in mammals (Lu et al., 2016). Since *Drosophila* encodes only one JNK gene, *bsk*, in contrast to ten JNK isoforms in mammalian cells, *Drosophila* lymph gland could serve as a simple model system to investigate *p53*-independent and *bsk*-dependent cell death pathways.

In addition to apoptotic cell death, overexpression of *tefu* in CZ resulted in loss of progenitor population, generating lymph glands containing only Hml>GFP-positive CZ (Fig. 5A, middle panel; Fig. S7). A similar phenotype has been reported when CZ cell death is induced by Hid/Reaper expression (Mondal et al., 2011). This resulted in proliferation of progenitors that are normally quiescent at 3L, followed by differentiation, eliminating progenitor population due to differentiation (Mondal et al., 2011). Although the expression of *bsk^DN^* alone in CZ did not affect differentiation (Fig. S7), co-expression of *bsk^DN^* and *tefu* significantly attenuated the differentiation phenotype induced by *tefu* expression (relative CZ area in the lymph gland: 99.4% in *Hml>tefu^GS13617^* versus 15.7% in *Hml>tefu^GS13617^*, *>bsk^DN^*) (Fig. 5A, third panel; Fig. S7). This result further supports the hypothesis that cell death in CZ caused by *tefu-bsk* signaling was responsible for the differentiation phenotype.

In agreement with our data, irradiation of mice revealed that resistance to IR-induced cell death correlates with differentiation status, showing more sensitivity in more differentiated cells: hematopoietic stem cells < common myeloid progenitors < granulocyte/macrophage progenitors (Mohrin et al., 2010). Irradiation induces apoptosis in all three cell populations in an ATM- and p53-dependent manner, suggesting that DDR in hematopoietic progenitors is conserved in *Drosophila*. In summary, we found that hematopoietic progenitors and differentiated hemocytes in the late 3L lymph gland undergo apoptotic cell death after irradiation. Different genes were required to induce cell death: *tefu*, *lok*, *p53*, and *reaper* in progenitors and *tefu*, *bsk*, and *reaper* in mature hemocytes. Along with previous reports, various cellular responses, such as survival (Xing et al., 2015), premature differentiation (Wagle and Song, 2020), and death (current study), are induced after irradiation in *Drosophila* stem cells, making this genetically tractable organism to be used as a valuable model system to further unravel the mechanism of distinct cellular responses of stem cells in response to DNA damage.

## MATERIALS AND METHODS

### *Drosophila* strains

All *Drosophila* fly stocks were maintained at 25°C with cornmeal, dextrose, and yeast medium. Canton S was used as the wild type. The following *Drosophila* stocks were used in this study: *p53^5A-1-4^* (BL6815), *hs-I-CreI* (BL6937), *UAS-DN-bsk* (BL6409), and *UAS-bsk* (BL9310); they were obtained from the Bloomington *Drosophila* Stock Center (Bloomington, IN, USA). *Tep4-Gal4* and *tefu^GS13617^* (DGRC204829) were obtained from the National Institute of Genetics (Japan) and the Kyoto Stock Center (Japan), respectively. Other fly stocks were: *lok^P6^* (W. Theurkauf), *hid^05014^* (H. Steller), *rpr^87^* (K. White), *UAS-puc* (M. Peifer), *dome-Gal4* (M. Zeidler), *Hml-Gal4* (S. Sinenko), *tefu^e00198^* (Exelixis, Boston, MA, USA) (Song et al., 2004).

### Immunohistochemistry

To obtain 3L, eggs were collected for 4 h, and the adult flies were removed from the cage. Hatched larvae were discarded at 23.5 h after adult removal, and newly hatched larvae were collected for 4 h and transferred to regular media until treatment. Late 3L at 88±2 h after egg hatching (AEH) were mock-treated, irradiated in a Cs^137^ gamma-irradiator, or heat-shock treated at 37°C for the indicated time in the water bath. Lymph glands were dissected and stained 4 h after treatment, as previously described (Evans et al., 2014). Briefly, the dissected lymph glands were fixed with 4% formaldehyde in 1X PBS for 20 min. After washing in 0.4% PBT (0.4% Triton-X 100 in PBS), samples were blocked with 10% normal goat serum in 0.1% PBT for 1 h. Samples were treated with rabbit anti-cleaved Dcp-1 (Asp216) (Cell Signaling Technology, Danvers, MA, USA, 1:250) or rabbit anti-Peroxidasin (Pxn, 1:2500) (Yoon et al., 2017) at 4°C overnight, washed with 0.4% PBT, and incubated with goat anti-rabbit Alexa Fluor 568 (Molecular Probes, Waltham, MA, USA, 1:400) for 2 h. After washing with 0.4% PBT, the tissues were stained with DAPI and mounted in 0.5% n-propyl gallate dissolved in glycerol.

TUNEL staining was performed using ApopTag Red *In Situ* Apoptosis Detection Kit (Millipore, Burlington, MA, USA) as previously described (Shim et al., 2014) with slight modification. The dissected lymph glands were fixed in 4% formaldehyde for 20 min and washed with 0.4% PBT. The samples were permeabilized with 0.1% sodium citrate in 0.1% Triton X-100 for 20 min on ice and incubated in an equilibration buffer for 1 h. After incubating in a reaction mixture overnight at 37°C in a humid chamber, the reaction was terminated by incubation in a stop reaction mix for 3 h at 37°C. The samples were washed in 0.4% PBT and blocked in 0.4% PBT containing 10% normal goat serum for 1 h. The samples were incubated with a rhodamine-conjugated anti-DIG antibody at 4°C overnight. After washing, the samples were stained with DAPI and mounted. All samples were visualized using a confocal laser scanning microscope (LSM 700, Carl Zeiss, Oberkochen, Germany).

### Quantification of samples

For quantitation, *Tep4-Gal4* or *dome-Gal4* (*dome^Meso^-Gal4* in the case of *lok* mutant)-driven GFP and *Hml-Gal4*-driven GFP (Pxn staining in the case of *tefu* and *reaper* mutants) were used as markers for MZ (Tep4/dome>GFP-positive, Hml>GFP-negative, or Pxn-negative) and CZ (Tep4/dome>GFP-negative, Hml>GFP-positive, or Pxn-positive), respectively. The total number, or total size, of the lobes was measured using DAPI staining. Images were processed using ImageJ (NIH), and the total number of cells, or total area, of each compartment was determined as described previously (Shim et al., 2012). Briefly, the GFP- or DAPI-positive areas were recalibrated into an identical threshold using the Binary tool. The area with an identical threshold was automatically captured using the Wand tool. The number of DAPI-positive cells in that area was counted using the Image-based Tool for Counting Nuclei (ITCN), and the size was measured using the Measure tool. The area, or the number of cells, in GFP- or Pxn-negative compartments was obtained by subtracting those in GFP- or Pxn-positive compartments from the whole lobe. The percentage of cell death was calculated as the number of cDcp-1 positive cells compared to the total number of DAPI-stained cells in the CZ or MZ. The cell number was the average of cell numbers in the three middle confocal sections (1.5 µm interval).

At least ten lymph glands from a minimum of two independent experiments were analyzed for each sample. All statistical analyses were performed using the GraphPad Prism software. The statistical significance of differences between two experimental samples was determined using an unpaired *t*-test with Welch's correction. Differences were considered statistically significant at *P*<0.05.

## Supplementary Material

Supplementary information
