# Supplementary material for: Chk2-p53 and JNK in irradiation-induced cell death of hematopoietic progenitors and differentiated cells in Drosophila larval lymph gland
Source: Biol Open. 2021 Aug 23;10(8):bio058809. doi: 10.1242/bio.058809 (PMC8411456; doi:10.1242/bio.058809)
Supplement: Supplementary information [file biolopen-10-058809-s1.pdf]

A

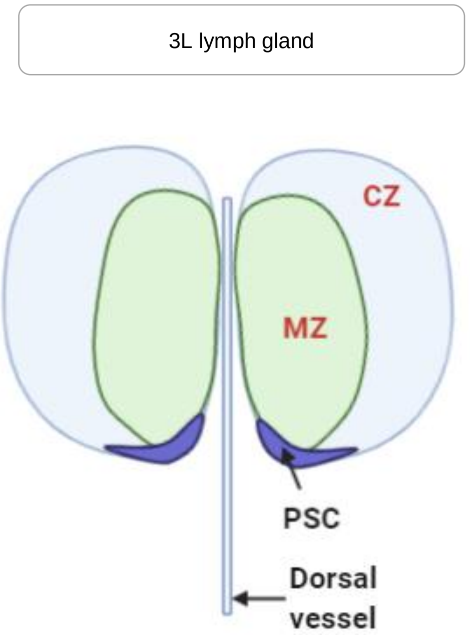

B

**MZ (medullary zone)**	**CZ (cortical zone)**
stem-like progenitors	differentiated cells
**Marker and driver system**:	**Marker and driver system**:
• dome-Gal4, UAS-GFP	• Hml-Gal4, UAS-GFP
• dome-Meso-Gal4, UAS-GFP	
• Tep4-Gal, UAS-GFP	

Fig. S1. *Drosophila* larval lymph gland. (A) Schematic presentation of a pair of anterior primary lobes in a *Drosophila* larval lymph gland. The primary lobe located along the dorsal vessel consists of a medullary zone (MZ), cortical zone (CZ), and the niche, called the posterior signaling center (PSC). (B) The markers and drivers used in this study to label or overexpress genes in MZ and CZ.

Biology Open • Supplementary information

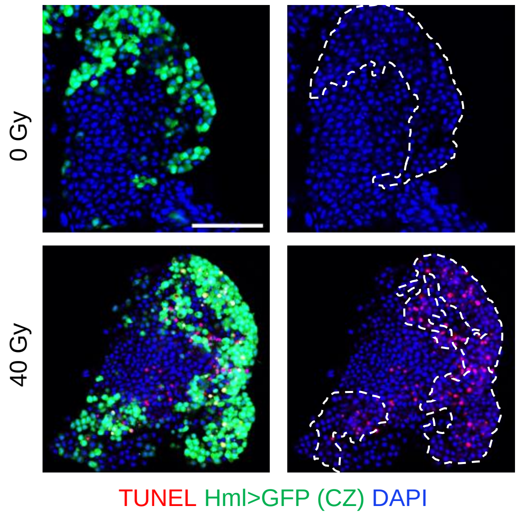

Fig. S2. Irradiation-induced apoptosis in the 3L lymph gland. The 3L were irradiated at 40 Gy and TUNEL staining was performed on the lymph glands 4 h after radiation treatment. The boundaries of CZ marked by Hml>GFP are indicated with white dotted lines. Scale bars, 50 μm

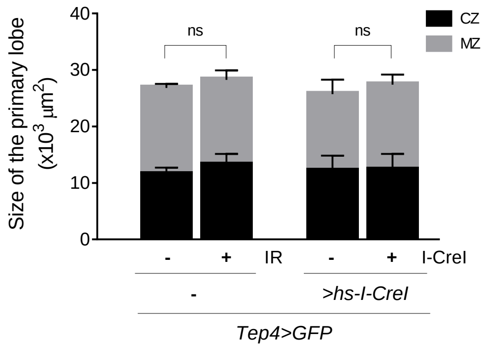

Fig. S3. DNA damage in the 3L lymph gland does not affect hematopoiesis. The size of the lymph glands and the proportion of MZ marked by Tep4>GFP from the lymph glands in Fig 1 are quantitated. The graph shows the average size and standard deviation from at least ten lymph glands. ns, not significant.

Biology Open • Supplementary information

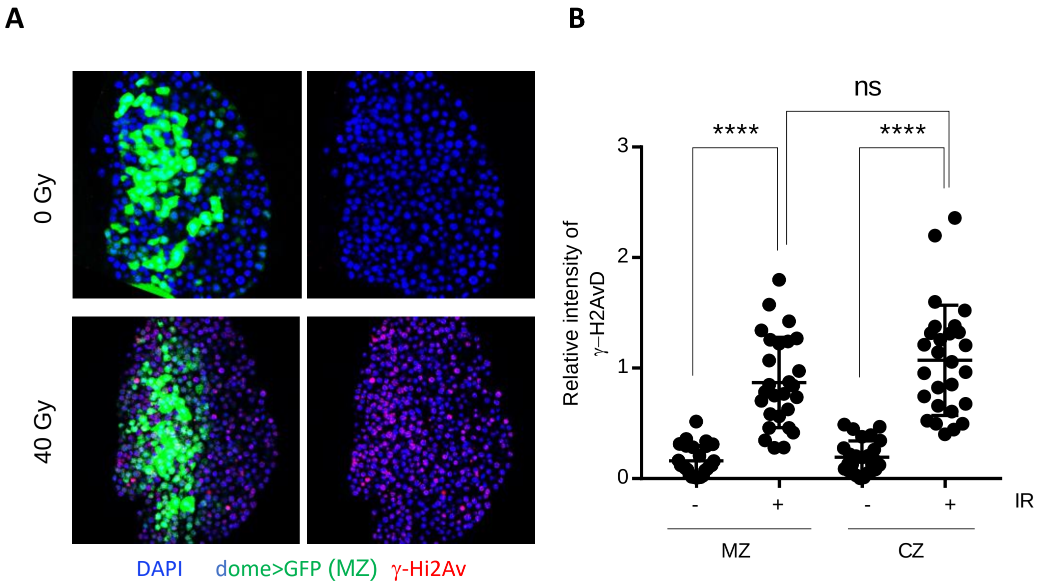

Fig. S4. The amount of DNA damage after IR in the 3L lymph gland. (A) The 3L was irradiated at 40 Gy, and the lymph gland was dissected and stained with γ-His2Av antibody (Rockland Inc, PA, 1:400) at 1 h post-irradiation. DAPI (blue), dome>GFP (green), and γ-His2Av (red) indicate DNA, progenitors, and double strand DNA breaks, respectively. (B) The relative intensity of the γ-His2Av signal in progenitors (MZ) and differentiated cells (CZ) with (+) and without (-) IR are shown. The values are normalized relative to the γ-His2Av intensity in 3L MZ after irradiation in each experiment. **** $p < 0.0001$; ns, not significant.

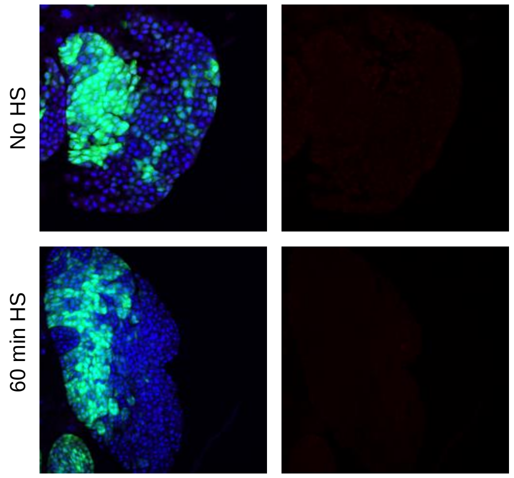

Fig. S5. Heat shock did not induce cell death in the wild type 3L lymph gland. The 3L expressing Tep4>GFP was treated with heat-shock. Four hours after treatment, the lymph gland was stained with cDcp-1 antibody. Scale bars, 50 μm. DAPI (blue), Tep4>GFP (green), and cDcp-1 (red) indicate DNA, progenitors, and apoptotic cells, respectively.

Biology Open • Supplementary information

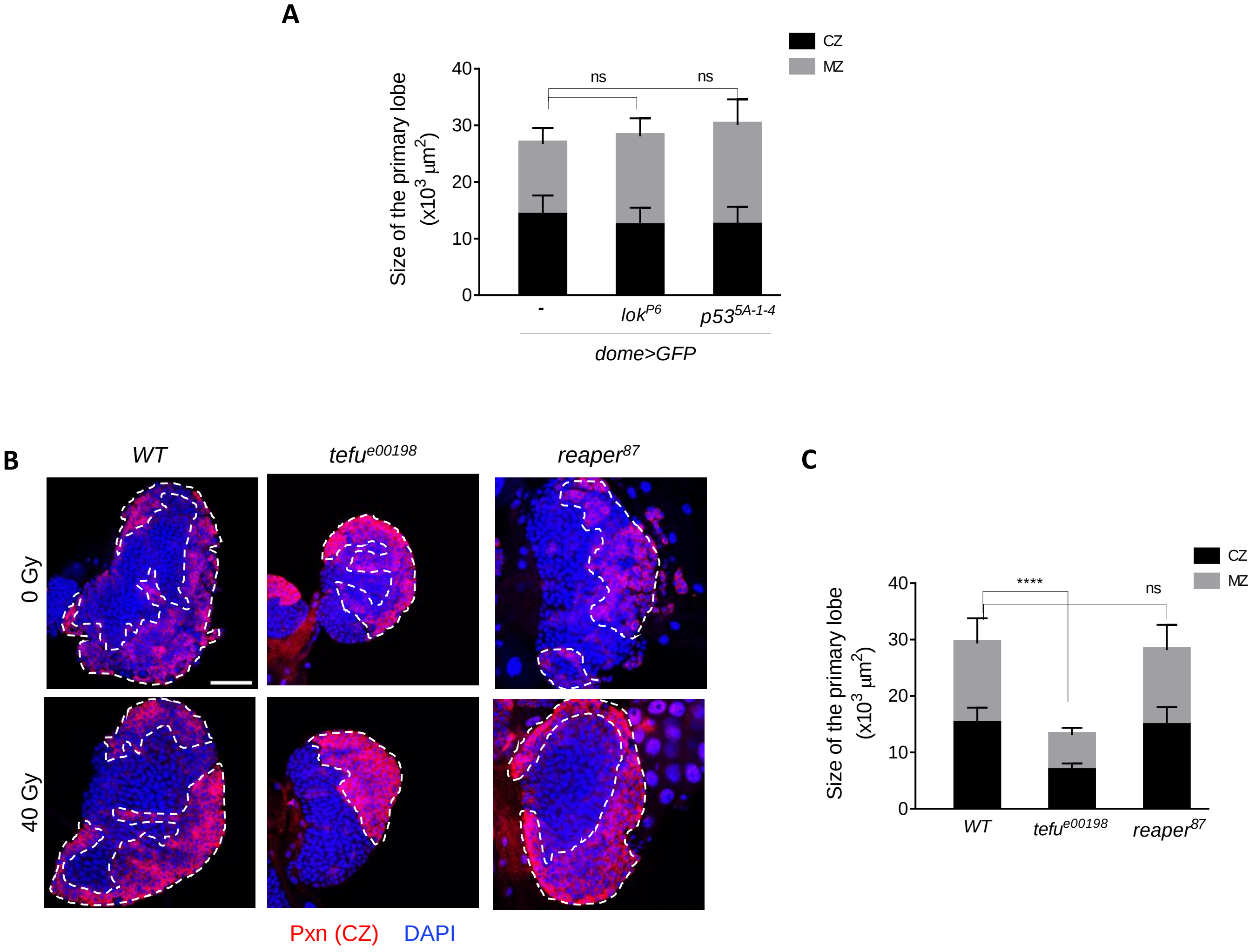

Fig. S6. Effect of irradiation on the hematopoiesis of 3L lymph gland in DDR mutants. (A) The size of the primary lobe and MZ marked by dome>GFP in the absence of irradiation in Fig 3B are quantitated. (B, C) The 3L of wild type, *tefu*^{e00198}, and *rpr*^{87} mutants were irradiated at 40 Gy. At 4 h post-irradiation, the lymph glands were stained using Peroxidasin (Pxn, red) to mark CZ. Scale bar, 50 µm. The boundary of CZ is marked with white dotted lines. (C) The size of the primary lobe and CZ marked by Pxn in the absence of irradiation are quantitated. **** $p < 0.0001$; ns, not significant.

Biology Open • Supplementary information

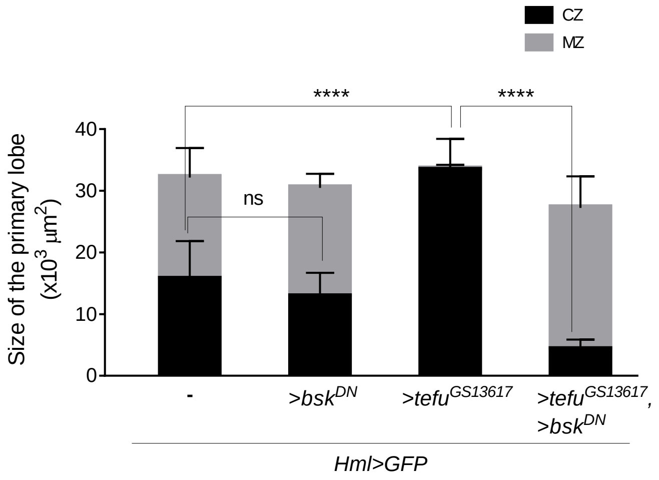

Fig. S7. Hematopoiesis of 3L lymph gland is regulated by overexpression of *tefu* in CZ. The size of the primary lobe and CZ marked by Hml>GFP in the absence of irradiation in Figs. 4, 5 are quantitated. **** $p < 0.0001$; ns, not significant.

Biology Open • Supplementary information
